# Pancreatic Cancer Presenting as a Pancreatic Duct Disruption

**DOI:** 10.1155/2019/6381249

**Published:** 2019-05-26

**Authors:** Annie Liu, Herbert J. Zeh, Brian A. Boone

**Affiliations:** ^1^Department of Neurobiology, University of Pittsburgh School of Medicine, Pittsburgh, PA, USA; ^2^Department of Surgery, University of Texas Southwestern Medical Center, Dallas, TX, USA; ^3^Department of Surgery, West Virginia University, Morgantown, WV, USA

## Abstract

The high mortality rate associated with pancreatic cancer necessitates accurate and early detection methods. Computed tomography currently is the primary diagnostic modality used; however, subtle imaging features in concert with novel clinical presentations may obscure the initial diagnosis. Here, we describe a unique initial presentation of pancreatic cancer as a pancreatic leak, with subtle initial CT evidence of malignancy. An 83-year-old female with prior surgical history of open splenectomy and ventral hernia repair presented with two weeks of vague abdominal pain and leukocytosis. Initial CT revealed abdominal peripancreatic fluid collections. Interventional radiology-guided drain placement was performed, which revealed amylase-rich pancreatic fluid within the collections. Repeat CT scan revealed subtle pancreatic duct dilation with slow resolution of the fluid collections. Ultimately, endoscopic ultrasound identified an ill-defined pancreatic mass, revealed to be pancreatic adenocarcinoma. The patient subsequently underwent an open distal pancreatectomy. Diagnosis of pancreatic cancer relies heavily on cross-sectional imaging, with no screening tests currently available. However, subtle radiographic features and unique clinical presentations may delay accurate diagnosis and staging. EUS may be a useful tool for initial evaluation of high-risk individuals.

## 1. Introduction

Pancreatic cancer has one of the lowest five-year survival rate of all malignancies (8.5%), accounting for 10.9 out of 100,000 deaths in the US [1]. Thus, accurate diagnosis and staging are important for improving mortality and morbidity. Computed tomography (CT) is the primary recommended method for initial screening for patients with high clinical suspicion of disease. In comparison to magnetic resonance imaging (MRI), biphasic CT is more cost-effective and widely available, has higher spatial resolution, has similar sensitivities for cancer detection, and thus remains the primary initial imaging modality used [2–4]. When clinical suspicion for pancreatic cancer is high despite negative initial CT or MRI, endoscopic ultrasound (EUS) is a reliable, accurate diagnostic modality with high sensitivity, especially for detecting early lesions [5, 6].

Here, we describe an unusual case of pancreatic adenocarcinoma initially presenting as pancreatic duct disruption in a patient with a prior splenectomy. Initial CT did not suggest pancreatic cancer; however, EUS was eventually used to evaluate subtle pancreatic duct dilation and revealed the presence of pancreatic adenocarcinoma. Our experience demonstrates that EUS is an accurate modality for detecting pancreatic cancer with both an initial negative CT and a complex clinical presentation.

## 2. Case

**Figure 1:**
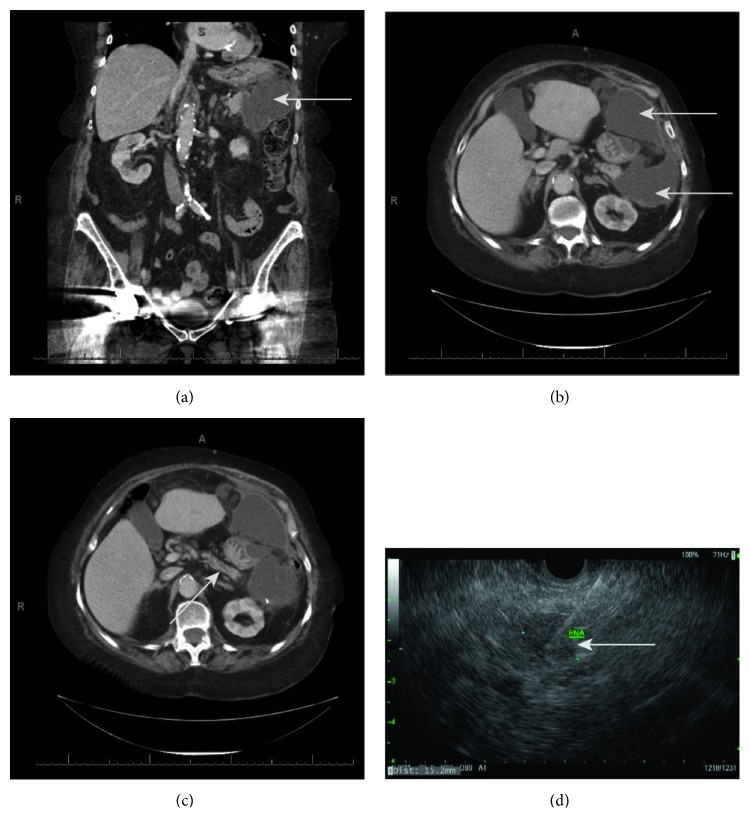
(a) Coronal section from initial CT showing large fluid collection (gray arrow) adjacent to the tail of the pancreas. (b) Axial section from initial CT showing anterior and posterior aspects of large fluid collection (gray arrows) surrounding the stomach and adjacent to the tail of the pancreas. (c) Axial section from initial CT showing mildly dilated pancreatic duct (gray arrow). (d) Still frame from EUS showing FNA needle within the visualized pancreatic mass (gray arrow).

An 83-year-old female presented to the emergency department with two weeks of vague abdominal pain. Her past medical history was significant for open splenectomy for spontaneous rupture three years prior to presentation and subsequent ventral hernia repair with mesh. She denied history of pancreatitis, diabetes mellitus, nor family history of gastrointestinal disease or malignancy. She was found to have a UTI and leukocytosis of 20,000, with LFTs and lipase within normal limits. Initial CT demonstrated abdominal fluid collections around the stomach and pancreatic tail, extending to segment two of the liver (Figures 1(a)–1(c)). She was subsequently admitted and treated with IV piperacillin-tazobactam for her UTI. On hospital day (HD) 2, she underwent IR-guided drain placement for percutaneous drainage of the abdominal fluid collection—aspirate gram stain revealed only scant WBCs and culture grew no organisms. The aspirate contained elevated amylase (>15,000 IU/L), suggesting pancreatic leak. Repeat CT revealed continued abdominal fluid collections requiring drain repositioning—ultimately three drains were placed to achieve adequate drainage. She was discharged and subsequently returned to the emergency room 23 days after initial presentation with nausea, abdominal discomfort, and persistent leukocytosis. Repeat CT revealed air and an enlarging fluid collection around one of her abdominal drains, which required IR-guided drain replacement. She was then started empirically on IV piperacillin-tazobactam. Analysis of the abdominal fluid cultures grew gram-negative rods. Repeat evaluation of her initial CT demonstrated potential pancreatic duct dilation in the mid pancreas (Figure 1(b)), and an EUS was performed to evaluate for abnormalities that may have precipitated the initial pancreatic leak. EUS revealed an ill-defined 17 mm × 10 mm mass in the body of the pancreas—an EUS-guided shark core aspiration of the mass was positive for adenocarcinoma (Figure 1(d)). Serum CA19-9 was 11.1 U/mL and serum CEA was 5.5 ng/mL. She subsequently underwent an open distal pancreatectomy with pathology demonstrating a stage pT3N1 17 mm invasive moderately differentiated ductal adenocarcinoma of the pancreatic body, in addition to pancreatic intraepithelial neoplasia. Pathology was positive for perineural invasion and lymphatic invasion, with negative proximal pancreatic and retroperitoneal margins. Immunohistochemistry revealed negative ALK and PDL-1 expression and preserved MLH1, MSH2, MSH6, and PMS2 expression. Her postoperative course was uncomplicated, and she was discharged on POD 20 to a skilled nursing facility.

## 3. Discussion

Here, we describe a diagnosis of pancreatic cancer in an 83-year-old female patient characterized by a unique clinical presentation and equivocal initial radiologic findings. EUS revealed the ill-defined pancreatic mass, and EUS-guided biopsy confirmed pancreatic adenocarcinoma. Given the high morbidity and mortality of this disease, evaluation and improvement of current diagnostic techniques is crucial.

Initial clinical symptoms of pancreatic cancer are vague and nonspecific, and patients typically present with abdominal pain and weight loss [7]. Painless jaundice is classically associated with pancreatic cancer. However, multiple studies report that only ~20% of patients present with jaundice [8, 9]. One study of 209 patients with histologically confirmed pancreatic cancer demonstrated that jaundice was significantly more likely to be a symptom in patients with a tumor in the pancreas head (93% of patients with jaundice) compared to a tumor located in the pancreas body or tail (8% and 0% of patients with jaundice, respectively) [8]. Additionally, 0.9-3.6% of patients presenting with acute pancreatitis are found to have pancreatic cancer [10]. A longitudinal study of almost 50,000 patients in a multiethnic cohort demonstrated a statistically significant relationship between a recent diagnosis of diabetes mellitus and subsequent diagnosis of pancreatic cancer within three years, suggesting that recent-onset diabetes may be a strong indication for pancreatic cancer screening [11]. The patient described here presented with vague abdominal pain and multiple peripancreatic fluid collections. She did not exhibit classic symptomology, nor did she have common risk factors for pancreatic cancer, such as diabetes mellitus, family history of GI malignancies, and chronic or acute pancreatitis. To our knowledge, this is the first description in the literature of pancreatic cancer presenting as a pancreatic duct disruption with a peripancreatic fluid collection.

CT, the recommended first line imaging modality for pancreatic cancer evaluation, has high sensitivity for large lesions and is useful in disease staging [12]. Recent studies demonstrate that EUS is potentially equal or even superior to CT in terms of initial disease detection [13–15]. EUS is both invasive and operator dependent, and CT is thus more cost-effective, widely available, and efficient. However, given its increased sensitivity for detecting early lesions, EUS provides valuable information for disease evaluation in patients with high clinical suspicion for pancreatic cancer and initial negative CT results [16, 17]. EUS is also potentially superior to CT in terms of determining lesion size and location, as well as local lymph node involvement [15]. Lastly, EUS allows for biopsy of visualized masses. More studies are necessary to determine if EUS is appreciably superior to CT or MRI for both initial diagnosis and disease staging. With improvement in the understanding of pancreatic cancer biomarkers, EUS may become appropriate for initial screening in high-risk individuals, as the technique is comparable in diagnostic accuracy to both CT and MRI while allowing immediate biopsy of identified lesions [18].

## 4. Conclusion

As demonstrated in this case of pancreatic cancer presenting initially as peripancreatic fluid collection, an atypical clinical presentation coupled with negative initial imaging studies may obscure the underlying diagnosis of pancreatic cancer. EUS has high diagnostic accuracy and allows for immediate tissue biopsy. Thus, EUS should be rigorously evaluated to determine its potential use in the initial screening of patients with high clinical suspicion for pancreatic cancer.
